# *In vivo *optical imaging of integrin α_V_-β_3 _in mice using multivalent or monovalent cRGD targeting vectors

**DOI:** 10.1186/1476-4598-6-41

**Published:** 2007-06-12

**Authors:** Zhao-Hui Jin, Véronique Josserand, Stéphanie Foillard, Didier Boturyn, Pascal Dumy, Marie-Christine Favrot, Jean-Luc Coll

**Affiliations:** 1 INSERM, U823, Cibles Diagnostiques ou Thérapeutiques et Vectorisation des Drogues dans le Cancer du Poumon, Institut Albert Bonniot, 38706 La Tronche Cedex, France; 2 Université Joseph Fourier, 38041 Grenoble Cedex 9, France; 3 CNRS, UMR5616, Ingénierie Moléculaire et Chimie des Composés Bio-organiques, LEDSS, 38041 Grenoble Cedex 9, France

## Abstract

**Background:**

The cRGD peptide is a promising probe for early non-invasive detection of tumors. This study aimed to demonstrate how RAFT-c(-RGDfK-)_4_, a molecule allowing a tetrameric presentation of cRGD, improved cRGD-targeting potential using *in vivo *models of α_V_β_3_-positive or negative tumors.

**Results:**

We chose the human embryonic kidney cells HEK293(β_3_) (high levels of α_V_β_3_) or HEK293(β_1_) (α_V_β_3_-negative but expressing α_V _and β1) engrafted subcutaneously (s.c.) in mice. Non-invasive *in vivo *optical imaging demonstrated that as compared to its monomeric cRGD analogue, Cy5-RAFT-c(-RGDfK-)_4 _injected intravenously had higher uptake, prolonged retention and markedly enhanced contrast in HEK293(β_3_) than in the HEK293(β_1_) tumors. Blocking studies further demonstrated the targeting specificity and competitive binding ability of the tetramer.

**Conclusion:**

In conclusion, we demonstrated that Cy5-RAFT-c(-RGDfK-)_4 _was indeed binding to the α_V_β_3 _receptor and with an improved activity as compared to its monomeric analog, confirming the interest of using multivalent ligands. Intravenous injection of Cy5-RAFT-c(-RGDfK-)_4 _in this novel pair of HEK293(β_3_) and HEK293(β_1_) tumors, provided tumor/skin ratio above 15. Such an important contrast plus the opportunity to use the HEK293(β_1_) negative control cell line are major assets for the community of researchers working on the design and amelioration of RGD-targeted vectors or on RGD-antagonists.

## Background

The tripeptide sequence Arg-Gly-Asp (RGD) [1,2] is a well known motif recognizing and interacting with integrin, a family of transmembrane heterodimeric glycoproteins composed of one α and one β subunits [3,4]. The structure of a cyclic pentapeptide containing RGD was optimized in order to provide a high affinity and selectivity for the α_V_β_3 _integrin [5], an integrin overexpressed at the surface of activated endothelial cells during angiogenesis [6,7] and in various types of tumor cells [8-11]. Radiolabeled cRGD peptides in combination with nuclear imaging techniques such as positron emission tomography (PET) and single photon emission computed tomography (SPECT) have been extensively studied for imaging of α_V_β_3 _expression in experimental tumors [12]. More recently, the development of *in vivo *optical imaging techniques and of various fluorescent-cRGD conjugates were also described for imaging cancer in mice [12-18]. In addition, it was shown that presenting multiple copies of the cRGD motif was usually associated with improved properties of the probes [16,19]. In this aim, our group has developed a novel tetrameric molecule by grafting four copies of cRGD onto a cyclic decapeptide platform called RAFT (Regioselectively Addressable Functionalized Template) [17,18,20]. When injected intravenously in nude mice bearing s.c. human ovarian carcinoma IGROV1 tumors, expressing a low level of α_V_β_3_, cyanine 5-labeled RAFT-c(-RGDfK-)_4 _showed a better tumor contrast than its monomeric analog [18].

In the present study, we took advantage of a particular tumor model for addressing RGD-mediated targeting specificity *in vivo*. This model derived from the naturally α_V_-positive and β_3_-negative HEK293 cell line was initially transfected by a plasmid encoding the human β_3 _chain, forming a strongly α_V_β_3_-positive HEK293(β_3_) stable clone. In addition, HEK293(β_1_), an α_V_β_3_-negative control overexpressing the β_1 _chain instead of the β_3_, had been also established. As their parent cell line HEK293, we show that the 2 β_3 _or β_1 _subclones are forming tumors when injected subcutaneously into athymic nude mice. Using these tumor models, our different RGD-based molecules and competition experiments, we demonstrate the extremely good specificity and improved tumor accumulation and retention of the Cy5-labeled RAFT-c(-RGDfK-)_4 _probe as compared to its monomeric analog. Since RGD-based antiangiogenic therapies are currently under investigation, and that cRGD can also serve as a ligand in human nuclear medicine, optimization of its specificity and drug delivery properties is of major importance for clinical applications.

## Results

### In vitro binding studies

**Figure 1:**
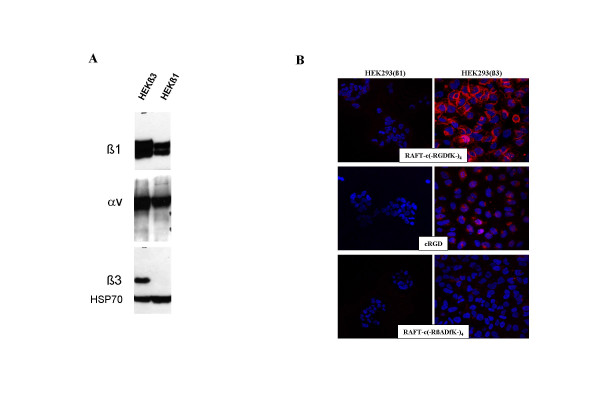
(A) Western blot analysis of expressions of integrin subunits α_V_, β_1 _and β_3 _in HEK293(β_1_) and HEK293(β_3_) cell lines. (B) Confocal laser scanning microscopic images of HEK293(β_1_) and HEK293(β_3_) cells incubated for 30 min at 37°C in the presence of 0.1 μM Cy5-labeled RAFT-c(-RGDfK-)_4_, cRGD, or RAFT-c(-RβADfK)_4_. Nuclei were stained with Hoechst 33342 (blue), and fluorescence signal from Cy5 was pseudocolored red. Original objective: Plan-Neofluar 40x/1.30 Oil ph3.

HEK293(β_3_) and HEK293(β_1_) cells are stable transfectants of human β_3 _and β_1 _subunit, respectively, from the human embryonic kidney cell line. Western blot analysis showed that α_V _was strongly expressed in both cell lines, and confirmed the successful transfection of β_3 _or β_1 _subunits (Fig 1A). This phenotype was also confirmed by FACS analysis performed with the anti-human α_V_β_3 _antibody [18]. These 2 cell lines, were then observed using confocal laser scanning microscopy (CLSM) after incubation with Cy5-labeled RAFT-c(-RGDfK-)_4_, cRGD, or RAFT-c(-RβADfK-)_4_. As shown in Fig. 1B, none of these peptides bound to the HEK293(β_1_) cells. As expected also, the RAFT-c(-RβADfK-)_4 _control peptide did not bind to the α_V_β_3_-positive HEK293(β_3_) cells. In contrast, cRGD and RAFT-c(-RGDfK-)_4 _were reacting with HEK293(β_3_) cells moderately and very strongly, respectively.

### Establishment of paired α_V_β_3_-positive and α_V_β_3_-negative tumor models

**Figure 2:**
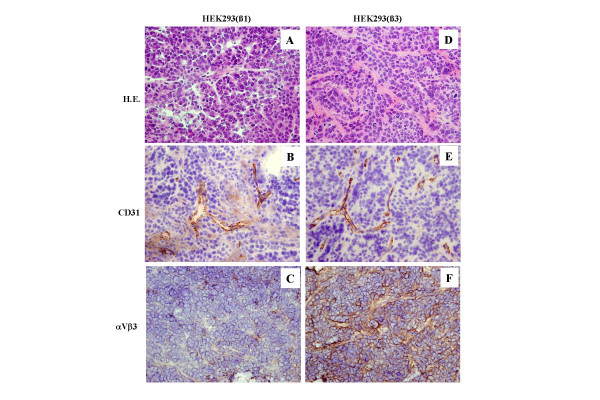
Hematoxylin-eosin (H.E.) staining and immunohistochemical staining with anti-CD31 and anti-α_V_β_3 _in HEK293(β_1_) or HEK293(β_3_) s.c. xenografts. Original magnification: × 20 objective lenses.

A s.c. inoculation of HEK293(β_3_) or HEK293(β_1_) cells in nude mice lead to tumor formation. This suggested that overexpression of β_3 _or β_1 _did not modify the known tumorigenicity of the parental HEK293 cell line (see ATCC number CRL-1573). Histological examination with hematoxylin and eosin (H.E.) staining shows that either HEK293(β_3_) or HEK293(β_1_) xenografts are composed of nodular cell masses and stroma (Fig 2). Immunohistochemical labeling of tumor sections shows positive α_V_β_3 _staining in HEK293(β_3_) cells but not in HEK293(β_1_) and a similar low to moderate vascularization as indicated by the CD 31-labeling of both tumors. Thus expression of the β3 chain was not lost during tumor growth and was not affecting the tumor vasculature.

### Whole body optical imaging

**Figure 3:**
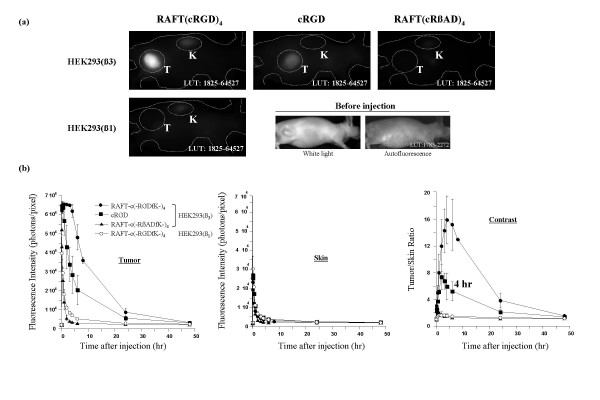
(a) Representative fluorescence images of Swiss nude mice bearing HEK293(β_1_) or HEK293(β_3_) s.c. tumors after i.v. injection of 10 nmol Cy5-labeled RAFT-c(-RGDfK-)_4_, cRGD, or RAFT-c(-RβADfK)_4_. All images were displayed at the indicated LUT (look-up-table) value. The values shown in each image represent the range of minimal to maximum signal intensity. T and K indicate tumor and kidney, respectively. (b) Time-courses of fluorescence intensities in tumors and skin as well as ratios of tumor *vs*. skin after i.v. injection of 10 nmol Cy5-labeled RAFT-c(-RGDfK-)_4_, cRGD, or RAFT-c(-RβADfK-)_4_. Solid circles: HEK293(β_3_) + Cy5-RAFT-c(-RGDfK-)_4_; Solid squares: HEK293(β_3_) + Cy5-cRGD; Solid triangles: HEK293(β_3_) + Cy5-RAFT-c(-RβADfK-)_4_; Open circles: HEK293(β_1_) + Cy5-RAFT-c(-RGDfK-)_4_. The fluorescence intensity was recorded as photons per pixel for a specified ROI. Data are expressed as means ± SD (n = 3–4).

Nude mice bearing s.c. tumor xenografts of HEK293(β_3_) or HEK293(β_1_) cell line received an i.v. injection of 10 nmol Cy5-labeled RAFT-c(-RGDfK-)_4_, cRGD, or RAFT-c(-RβADfK-)_4 _and were imaged at different time points during 2 days. As shown in Fig. 3a, four hours after injection a stronger tumor uptake was observed for RAFT-c(-RGDfK-)_4 _than for cRGD, while the control probe RAFT-c(-RβADfK-)_4 _was not retained in the tumor. Interestingly, the α_V_β_3_-negative HEK293(β_1_) tumors did not take-up the RAFT-c(-RGDfK-)_4 _peptide, demonstrating the specificity of RAFT-c(-RGDfK-)_4 _for the α_V_β_3 _integrin. The quantitative analysis also showed that the tetramer and the monomer reached similar maximal tumor uptake 5 to 30 min postinjection (p.i.) (Fig. 3b). Between 30 min an 4 hr p.i., the tetramer's signal remained very elevated in the tumor (65 472 ± 90 to 61 875 ± 3434 photons/pixel) while a marked decrease (from 63 744 ± 3 031 to 28 349 ± 9 727 photons/pixel) was measured with the cRGD. At later time points RAFT-c(-RGDfK-)_4 _always showed a better tumor accumulation than the monomer. The negative control probe RAFT-c(-RβADfK-)_4 _was rapidly washed-out from the tumors. In normal skin, all 3 probes exhibited similar kinetic curves, except at early time points (5 min to 1 hr) where cRGD showed a somewhat stronger non-specific diffusion (Fig. 3b). Finally, the tumor contrast (T/S ratio) was markedly enhanced with RAFT-c(-RGDfK-)_4_. Four hr p.i. the T/S ratio reached the value of 15.9 ± 3.6 with RAFT-c(-RGDfK-)_4_. This was significantly higher than that of the monomeric cRGD (5.9 ± 2.0), or the 1.4 ± 0.1 ratio obtained for the control probe. Importantly, RAFT-c(-RGDfK-)_4 _did not accumulate in the α_V_β_3_-negative HEK293(β_1_) xenografts. Indeed, the measured signal obtained with RAFT-c(-RGDfK-)_4 _in HEK293(β_1_) tumors was similar to that of RAFT-c(-RβADfK-)_4 _in HEK293(β_3_) tumors.

### Confocal microscopic observation of RGD-Cy5 conjugate distribution

**Figure 4:**
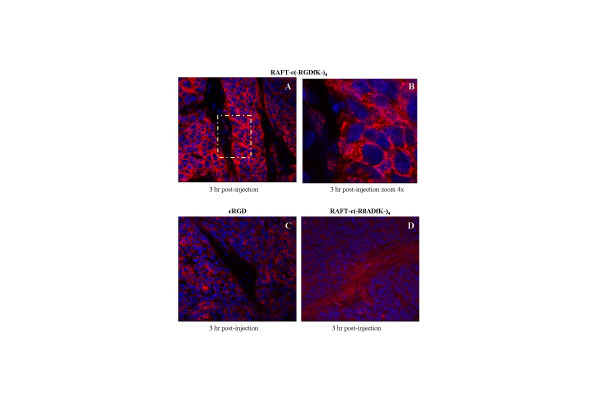
Confocal laser scanning microscopic images of HEK293(β_3_) s.c. tumors dissected at 3 hr after i.v. injection of 10 nmol Cy5-labeled RAFT-c(-RGDfK-)_4 _(A, B), cRGD (C), or RAFT-c(-RβADfK-)_4 _(D). Paraformaldehyde-fixed cryosections were incubated with Hoechst 33342 for nuclear staining (blue). Signal from Cy5 was pseudocolored red. Original objective: Plan-Apochromat 63x/1.4 Oil; an additional zoom of 4x was added for the insert in B.

Tumors of mice treated as mentioned above were excised 3 or 24 hr p.i, and analyzed by CLSM imaging (Fig. 4). Cy5-RAFT-c(-RGDfK-)_4 _was massively internalized by tumor cells as shown at a higher magnification in the insert (Fig. 4B). While virtually each tumor cell was strongly labeled at 3 hr, it was still easily detectable in a large proportion of tumor cells after 24 hr (data not shown). A similar pattern was obtained with the monomeric cRGD although the intensity of the signal was lower (Fig. 4C). No specific fluorescence was found with the control peptide Cy5-RAFT-c(-RβADfK-)_4 _(Fig 4D).

### Blocking study

**Figure 5:**
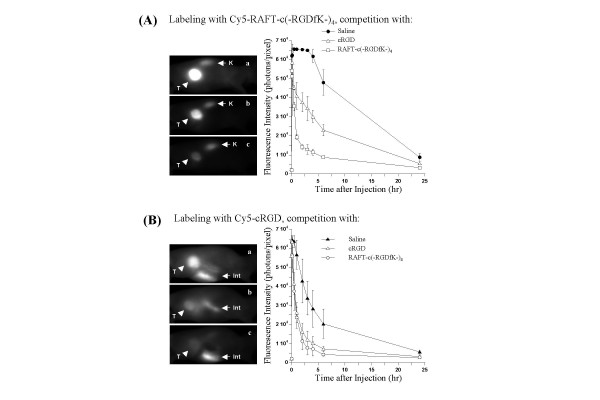
Blocking of Cy5-labeled RGD peptide accumulation in HEK293(β_3_) s.c. tumors by coinjection with unlabeled RGD peptide. **(A) **Tumor-bearing mice received i.v. injection of 10 nmol Cy5-RAFT-c(-RGDfK-)_4 _alone (a), or coinjected with 1200 nmol cRGD (b) or 300 nmol RAFT-c(-RGDfK-)_4 _(c). **(B) **Tumor-bearing mice received i.v. injection of 10 nmol Cy5-cRGD alone (a), or coinjected with 1200 nmol cRGD (b) or 300 nmol RAFT-c(-RGDfK-)_4 _(c). Left panel: representative fluorescence images at 2 hr p.i.; Right panel: kinetics of fluorescence intensities in tumors. T, K and Int indicate tumor, kidney and intestine, respectively. Data are expressed as means ± SD (n = 2–4).

In order to further establish the *in vivo *specificity of Cy5-RGD conjugates, 10 nmol Cy5-RAFT-c(-RGDfK-)_4 _or Cy5-cRGD were coinjected with 300 nmol unlabeled tetrameric RGD or 1200 nmol unlabeled monomeric cRGD. The differences in the injected doses of unlabeled molecules were calculated in order to maintain equal concentrations of the competing RGD motifs. As shown in Fig. 5A, the tumor uptake of Cy5-RAFT-c(-RGDfK-)_4 _was significantly reduced in the presence of ''cold'' (unlabeled) monomer and this effect was more obvious when the ''cold'' tetramer was used. As an example, at 3 hr p.i. the signal intensities were significantly decreasing (*p *< 0.0001) from 65 472 ± 80 without competitor to 34 339 ± 6 402 in the presence of ''cold'' cRGD (reduction of 50%) and down to 12 894 ± 2 504 when the ''cold'' tetramer was in excess (reduction of 80%). This blocking effect was obvious on the corresponding images (Fig. 5A, left panel). In addition, it is important to note that the strong decrease of the signal in the tumors was observed while the kidneys were showing identical intensities. This indicated that, as expected, the non-specific renal uptake of the tetrameric RGD was not affected by the presence of the different competitors. Similarly, a reduction of at least 50 to 60 % was obtained when Cy5-cRGD was used for labeling (Fig. 5B). The blocking effect of both competitors was very strong even if RAFT-c(-RGDfK-)_4 _was slightly more efficient.

## Discussion

RGD-based peptides are certainly the most frequently used molecules for tumor targeting and are currently in use for selective drug delivery and tumor imaging in preclinical models or in clinical trials. In this study we present evidences that presenting four copies of the cRGD motif on our RAFT carrier greatly improves cRGD-mediated tumor targeting *in vivo *of α_V_β_3_-positive tumors.

*In vitro *and *in vivo*, the α_V_β_3_-positive HEK293(β_3_) cells and tumors were very strongly recognized by Cy5-RAFT-c(-RGDfK-)_4 _but not by the negative control RAFT-c(-RβADfK-)_4_. In addition, the α_V_β_3_-negative HEK293(β_1_) samples remained negative after staining with the RGD or RβAD-based peptides. Furthermore, fluorescence images of both cultured cells and excised tumors clearly demonstrate the stronger labeling of HEK293(β_3_) cells by Cy5-RAFT-c(-RGDfK-)_4 _as compared to its monomeric analogue, confirming the enhanced receptor binding achieved when multiple RGD motifs are presented by a single template. The tetrameric RGD exhibited also stronger signal intensity in tumors, longer retention and much better contrast as compared to its monomeric analogue. Such effects could be explained by its augmented receptor-binding affinity due to the polyvalency effect [17,19,20] and increased molecular size which certainly delays the circulation and tumor retention time of the Cy5-RAFT-c(-RGDfK-)_4_. Finally, the active internalization of the Cy5-RAFT-c(-RGDfK-)_4 _probe may also contribute to its improved accumulation in the tumor cells. As shown on the tumor sections, the internalization was very strong since most of the signal was coming from the cytoplasm of the target cells. This suggests that such vector could be highly efficient to deliver drugs intracellularly. Multivalent presentation of ligands is improving significantly the targeting of tumors and several highly efficient targeting molecules allowing a multivalent presentation of RGD have been described [21-27]. Nonetheless, for some of these molecules the chemical formulation is poorly characterized and thus the number of ligand motifs being added on a polymer is random and cannot be controlled. In addition, the conformation of these molecules is not constrained. It is thus impossible to separate spatially the different biological functions presented by a single molecule nor it is possible to know its exact structure. These problems are avoided using RAFT-c(-RGDfK-)4 because the chemistry we use is regio- and chemo-selective. Thus the synthesis and purification of the final molecules are perfectly controlled even at gram scale. In addition, the RAFT architecture allows a spatial separation between the targeting and "drug-delivery" domains. Finally, RAFT is also interesting because its geometry allows a presentation of four RGD motifs at a very high density on its small surface.

To further confirm the receptor binding specificity of the Cy5-labeled RGD tetramer, blocking experiments were performed *in vivo*. In agreement with other reports using the monomeric cRGD [13,14,16], we observed an almost complete inhibition of cRGD accumulation in the presence of an excess of "cold" cRGD or RAFT-c(-RGDfK-)_4_. More interestingly the opposite experiment showed that while cRGD was able to block roughly 50% of Cy5-RAFT-c(-RGDfK-)_4 _accumulation, the presence of an excess of unlabeled tetramer was reducing by more than 80% the Cy5-RAFT-c(-RGDfK-)_4 _signal in the tumor. Finally Cy5-RAFT-c(-RGDfK-)_4 _shows higher renal uptake than Cy5-cRGD. This was observed in either tumor-bearing or normal mice. This renal retention is likely to be non-specific since it was not modified by the presence of an excess of unlabeled tetramer or monomer.

HEK293(β_3_) and HEK293(β_1_) might be an interesting duo of tumors forming from cell lines which differ only by their integrin β_3 _status. One is α_V_β_3_-positive and the other is α_V_β_3_-negative. Another model, M21 was also described. The α_V_β_3_-positive M21 and its α_V_β_3_-negative variant M21-L human melanoma cell lines were the first reported paired models for *in vivo *evaluation of α_V_β_3 _receptor binding specificity of RGD peptides [13,28,29]. M21-L cells were selected and maintained as a stable variant of M21 unable to synthesize the α chain but with normal levels of the β chain [30]. Here, we present another duo of α_V_β_3 _positive and negative s.c. tumor xenografts. HEK293(β_3_) and HEK293(β_1_) cell lines are stable transfectants of the human embryonic kidney cell line HEK293, overexpressing the human integrin β_3 _and β_1 _subunits respectively. While the original HEK293 cells express high levels of α_V _but negligible levels of α5, β_3 _and β_1_, HEK293(β_3_) expresses impressive amounts of α_V_β_3_, and HEK293(β_1_) cells mainly form the α_V_β_1 _receptor (another known receptor of fibronectin and vitronectin). This model is of great interest for the *in vivo *study of RGD-based targeting vectors since tumor/skin ratio of more than 15 can be obtained. Such a large dynamic is allowing precise measurements of the impact of treatments or chemical modifications possibly affecting the RGD-mediated targeting. In addition, the negative control cell line is a major asset to confirm the specificity of these RGD-delivery systems.

## Conclusion

Using such paired tumor models, we demonstrated that RAFT-c(-RGDfK-)_4 _is specific for the α_V_β_3 _receptor and internalized. In addition, due to its multifunctional backbone, it can carry multiple biological functions on a single, spatially and chemically defined molecule. Finally, the production of large quantities of perfectly controllable batches makes of RAFT-c(-RGDfK-)_4 _a powerful and versatile synthetic vector for clinical applications like targeted-drug delivery or molecular imaging of cancer. Ultimately, our goal will be to combine these two applications and to use RAFT-c(-RGDfK-)_4 _for imaging and quantification of its targeted-drug delivery efficiency.

## Methods

### RGD-Peptides Synthesis and Fluorescent Labeling

The detailed protocol for synthesis of RGD peptides was reported previously [20]. Here, a brief description was given below for the strategy of RGD multimerization and fluorescence labeling. RAFT is a cyclic decapeptide (c [-Lys(Boc)-Lys(Alloc)-Lys(Boc)-Pro-Gly-Lys(Boc)-Lys(Alloc)-Lys(Boc)-Pro-Gly-]) with up to six lysine residues. Protection of the lysine in position 1, 3, 6, or 8 and of the two in positions 2 and 7 results in RAFT molecules having two orthogonally addressable domains pointing on either side of the cyclopeptide backbone. On the upper face, four copies of the c [-RGDfK-] peptide were grafted via an oxime bond (R1-O-N = C-R2) for recognition of the integrin. On the opposite side of RAFT, Cy5 mono NHS (N-hydroxysuccinimide) ester (Amersham Biosciences, Uppsala, Sweden) was added on the lysine chain (c [-KKKPGKAKPG-]) [17]. As a negative control probe, Cy5-labeled RAFT-c(-RβADfK-)_4 _was also synthesized in a similar way. Changing the G amino-acid by a β-Ala abolishes RGD-mediated affinity for the integrins. All the probes were dissolved in phosphate-buffered saline (PBS) for the *in vitro *and *in vivo *application.

### Cell Lines and Culture Conditions

HEK293(β_3_) and HEK293(β_1_) cells, stable transfectants of human β_3 _and β_1 _subunit, respectively, from the human embryonic kidney cell line (kindly provided by J-F. Gourvest, Aventis, France) were cultured in DMEM enriched with 4.5 g.L^-1 ^glucose and supplemented with 1% glutamine, 10% fetal bovine serum (FBS), 50 units/ml penicillin, 50 μg/ml streptomycin and 700 μg/ml Geneticin (G418 sulfate, Gibco, Paisley, UK). The 2 cell lines were cultured at 37°C in a humidified 95% air:5% CO_2 _atmosphere.

### Western blot analysis of integrin subunit expression

Cells were lysed in RIPA lysis buffer (50 mM Tris-HCl, pH 7.5, 150 mM NaCl, 0.5% sodium deoxycholate, 0.1% SDS, 1% Nonidet P-40, 1 mM NaF, 1 mM Na_3_VO_4_, 0.5 mM phenylmethylsulfonyl fluoride, 10 μg/ml for each of leupeptin, aprotinin, and pepstatin) for 30 min on ice, and then the lysates were centrifuged at 17000 × *g *for 15 minutes at 4°C. The protein concentration of the supernatant was quantified using a protein assay kit (Bio Rad Labs., Richmond, CA). Aliquots of protein (40 μg) were subjected to electrophoresis on 7–10% polyacrylamide gels containing 0.1% SDS, followed by electrophoretic transfer onto PVDF-membranes, Hybond™-P (Amersham Biosciences UK Limited, Little Chalfont, Buckinghamshire, UK). The membranes were then incubated with primary antibody: rabbit anti-human integrin α_V _polyclonal antibody (1:5000; Chemicon International, Inc., Temecula, CA), rabbit anti-integrin β_1 _tail serum (1:1500; kindly provided by Dr C. Albiges-Rizo, Grenoble, France) or mouse anti-human β_3 _monoclonal antibody (clone VI-PL2, 1:100; BD Biosciences PharMingen, San Diego, CA). To monitor equal protein loading, membranes were also probed for actin using rabbit anti-actin polyclonal antibody (1:1000; Sigma) or for HSP70 using mouse anti-HSP70 monoclonal antibody (1:5000; Affinity BioReagents Inc.). For visualization, horseradish peroxidase (HRP)-conjugated secondary antibodies, followed by ECL™ immunodetection (Amersham Biosciences UK Limited) were used.

### Animal, Tumor Models and Histochemistry

Animal procedures were in agreement with the EEC guidelines. Female athymic Swiss nude mice, purchased from Janvier (Le Genest Saint Isle, France) at 6–8 weeks of age were used and maintained under specific pathogen-free conditions. Subcutaneous (s.c.) injection of 20 × 10^6 ^HEK293(β_3_) or HEK293(β_1_) cells suspended in 200 μl of PBS into the right flank of mice resulted in formation of 6–8 mm-diameter tumors after 4–6 weeks. Immunostaining with mouse anti-human integrin α_V_β_3 _monoclonal antibody, clone LM609 (1:100; Chemicon) was performed on acetone-fixed cryosections using M.O.M. immunodetection (peroxidase) Kit (Vector laboratories, Inc., Burlingame, CA). Rat anti-mouse CD31 monoclonal antibody, clone MEC13.3 (1:3000; BD Biosciences PharMingen) staining was performed on methanol-fixed cryosections using Strept-. ABComplex/HRP immunodetection Kit (DakoCytomation). The nuclei were counterstained with hematoxylin.

### In Vitro Studies

Cells were seeded on sterilized 18-mm-diameter glass coverslips in 12-well plates (3 × 10^5 ^cells per well), and incubated overnight at 37°C. Afterwards, the cells were washed with PBS and incubated at 37°C in the presence of Cy5-labeled peptides RAFT-c(-RGDfK-)_4_, cRGD or RAFT-c(-RβADfK-)_4 _at final concentration of 0.1 μM for 30 min. They were then washed with PBS, fixed with 2% paraformaldehyde at room temperature for 10 min. The nuclei were stained with 5 μM Hoechst 33342, and the coverslips were inverted onto glass slides using Mowiol (Calbiochem, San Diego, CA) mounting medium. The slides were observed with a confocal laser scanning microscopy (CLSM) (LSM510, Zeiss, France).

### In Vivo Optical Imaging of Tumor-bearing Mice

The mice bearing s.c. HEK293(β_3_) or HEK293(β_1_) tumors at diameter of 6–8 mm were used for imaging experiments. They received intravenous (i.v.) injection of Cy5-labeled peptides RAFT-c(-RGDfK-)_4_, cRGD or RAFT-c(-RβADfK-)_4 _at 10 nmol for each mouse (n = 3–4 for each probe). For the blocking experiments, s.c. HEK293(β_3_) tumor-bearing mice (n = 2–4 for each group) received coinjection of Cy5 labeled RAFT-c(-RGDfK-)_4 _or cRGD (10 nmol/mouse) together with unlabeled RAFT-c(-RGDfK-)_4 _(300 nmol/mouse) or cRGD (1200 nmol/mouse). Four times higher molar concentration of cRGD was used than that of tetramer to have same number of cRGD motifs.

Fluorescence reflectance imaging was performed using a Hamamatsu optical imaging system described previously [17,18]. In brief, imaging was carried out in a dark box, and anesthetized animal was illuminated with a monochromatic 633 nm light (50 μW.cm^-2^). The re-emitted fluorescence was filtered using a colored glass filter RG 665 (optical density > 5 at the excitation wavelength 633 nm) and collected with a cooled (-70°C) digital charge-coupled device (CCD) camera (Hamamatsu digital camera C4742-98-26LWGS, Hamamatsu Photonics K.K., Japan). All fluorescence images were acquired using 100 ms of exposure time, with other related parameters kept constant throughout the experiment. Images were acquired as 16-bit TIFF files which can provide a dynamic of up to 65535 grey levels. Image processing used in this study, including setting LUT (look-up-table) range and measurement of the fluorescence intensity for each region of interest (ROI), were performed using the Wasabi software (Hamamatsu). It is also important to note that all the images are presented without background subtraction. For quantifying tumor contrast, the mean fluorescence intensities of the tumor area (T) and that of the distant skin area (S) were calculated; dividing T by S produced the ratio between tumor tissues and background level.

### Histological Distribution of RGD-peptides in Tumors

At 3 and/or 24 hr after i.v. injection of 10 nmol of Cy5-labeled RAFT-c(-RGDfK-)_4_, cRGD or RAFT-c(-RβADfK-)_4_, the mice were euthanized and tumors were excised, frozen in liquid nitrogen and stored at -80°C. Sections of 20–30 μm thickness were fixed with 2% paraformaldehyde at room temperature for 10 min. The nuclei were stained with 5 μM Hoechst 33342, and the coverslips were mounted using Mowiol and kept at 4°C in the dark until observation using CLSM.

### Statistical Analysis

All the data are given as mean ± standard (SD) of n independent measurements. Statistical analysis was performed using two-tailed nonparametric Mann-Whitney *t*-test. Statistical significance was assigned for values of *p *< 0.05.

## Authors' contributions

ZJ and JLC were in charge of the experiments. VJ ran the in vivo imaging. SF, DB and PD were in charge of the synthesis of the molecules, MCF and JLC designed the study. All authors read and approved the final manuscript.

## References

[B1] Ruoslahti E (1996). RGD and other recognition sequences for integrins. Annu Rev Cell Dev Biol.

[B2] Takagi J (2004). Structural basis for ligand recognition by RGD (Arg-Gly-Asp)-dependent integrins. Biochem Soc Trans.

[B3] Hynes RO (1987). Integrins: a family of cell surface receptors. Cell.

[B4] Hynes RO (1992). Integrins: versatility, modulation, and signaling in cell adhesion. Cell.

[B5] Haubner R, Gratias R, Diefenbach B, Goodman SL, Jonczyck A, Kessler H (1996). Structural and functional aspects of RGD-containing cyclic pentapeptides as highly potent and selective integrin αVβ3 antagonists. Journal of American Chemical Society.

[B6] Stromblad S, Cheresh DA (1996). Integrins, angiogenesis and vascular cell survival. Chem Biol.

[B7] Gladson CL (1996). Expression of integrin alpha v beta 3 in small blood vessels of glioblastoma tumors. J Neuropathol Exp Neurol.

[B8] Gladson CL, Cheresh DA (1991). Glioblastoma expression of vitronectin and the alpha v beta 3 integrin. Adhesion mechanism for transformed glial cells. J Clin Invest.

[B9] Gehlsen KR, Davis GE, Sriramarao P (1992). Integrin expression in human melanoma cells with differing invasive and metastatic properties. Clin Exp Metastasis.

[B10] Seftor RE, Seftor EA, Gehlsen KR, Stetler-Stevenson WG, Brown PD, Ruoslahti E, Hendrix MJ (1992). Role of the alpha v beta 3 integrin in human melanoma cell invasion. Proc Natl Acad Sci U S A.

[B11] Filardo EJ, Brooks PC, Deming SL, Damsky C, Cheresh DA (1995). Requirement of the NPXY motif in the integrin beta 3 subunit cytoplasmic tail for melanoma cell migration in vitro and in vivo. J Cell Biol.

[B12] Kwon S, Ke S, Houston JP, Wang W, Wu Q, Li C, Sevick-Muraca EM (2005). Imaging dose-dependent pharmacokinetics of an RGD-fluorescent dye conjugate targeted to alpha v beta 3 receptor expressed in Kaposi's sarcoma. Mol Imaging.

[B13] Wang W, Ke S, Wu Q, Charnsangavej C, Gurfinkel M, Gelovani JG, Abbruzzese JL, Sevick-Muraca EM, Li C (2004). Near-infrared optical imaging of integrin alphavbeta3 in human tumor xenografts. Mol Imaging.

[B14] Chen X, Conti PS, Moats RA (2004). In vivo near-infrared fluorescence imaging of integrin alphavbeta3 in brain tumor xenografts. Cancer Res.

[B15] Gurfinkel M, Ke S, Wang W, Li C, Sevick-Muraca EM (2005). Quantifying molecular specificity of alphavbeta3 integrin-targeted optical contrast agents with dynamic optical imaging. J Biomed Opt.

[B16] Cheng Z, Wu Y, Xiong Z, Gambhir SS, Chen X (2005). Near-infrared fluorescent RGD peptides for optical imaging of integrin alphavbeta3 expression in living mice. Bioconjug Chem.

[B17] Garanger E, Boturyn D, Jin Z, Dumy P, Favrot MC, Coll JL (2005). New multifunctional molecular conjugate vector for targeting, imaging, and therapy of tumors. Mol Ther.

[B18] Jin ZH, Josserand V, Razkin J, Garanger E, Boturyn D, Favrot MC, Dumy P, Coll JL (2006). Non-invasive optical imaging of ovarian metastases using Cy5-labeled RAFT-c(-RGDfK-)4.. Mol Imaging.

[B19] Wu Y, Zhang X, Xiong Z, Cheng Z, Fisher DR, Liu S, Gambhir SS, Chen X (2005). microPET imaging of glioma integrin {alpha}v{beta}3 expression using (64)Cu-labeled tetrameric RGD peptide. J Nucl Med.

[B20] Boturyn D, Coll JL, Garanger E, Favrot MC, Dumy P (2004). Template assembled cyclopeptides as multimeric system for integrin targeting and endocytosis. J Am Chem Soc.

[B21] Weissleder R, Kelly K, Sun EY, Shtatland T, Josephson L (2005). Cell-specific targeting of nanoparticles by multivalent attachment of small molecules. Nat Biotechnol.

[B22] Shukla R, Thomas TP, Peters J, Kotlyar A, Myc A, Baker Jr JR (2005). Tumor angiogenic vasculature targeting with PAMAM dendrimer-RGD conjugates. Chem Commun (Camb).

[B23] Schraa AJ, Kok RJ, Moorlag HE, Bos EJ, Proost JH, Meijer DK, de Leij LF, Molema G (2002). Targeting of RGD-modified proteins to tumor vasculature: a pharmacokinetic and cellular distribution study. Int J Cancer.

[B24] Harbottle RP, Cooper RG, Hart SL, Ladhoff A, McKay T, Knight AM, Wagner E, Miller AD, Coutelle C (1998). An RGD-oligolysine peptide: a prototype construct for integrin-mediated gene delivery. Hum Gene Ther.

[B25] Bibby DC, Talmadge JE, Dalal MK, Kurz SG, Chytil KM, Barry SE, Shand DG, Steiert M (2005). Pharmacokinetics and biodistribution of RGD-targeted doxorubicin-loaded nanoparticles in tumor-bearing mice. Int J Pharm.

[B26] Ye Y, Bloch S, Xu B, Achilefu S (2006). Design, synthesis, and evaluation of near infrared fluorescent multimeric RGD peptides for targeting tumors. J Med Chem.

[B27] Liu S, Hsieh WY, Jiang Y, Kim YS, Sreerama SG, Chen X, Jia B, Wang F (2007). Evaluation of a (99m)Tc-labeled cyclic RGD tetramer for noninvasive imaging integrin alpha(v)beta3-positive breast cancer. Bioconjug Chem.

[B28] Haubner R, Bruchertseifer F, Bock M, Kessler H, Schwaiger M, Wester HJ (2004). Synthesis and biological evaluation of a (99m)Tc-labelled cyclic RGD peptide for imaging the alphavbeta3 expression. Nuklearmedizin.

[B29] Li C, Wang W, Wu Q, Ke S, Houston J, Sevick-Muraca E, Dong L, Chow D, Charnsangavej C, Gelovani JG (2006). Dual optical and nuclear imaging in human melanoma xenografts using a single targeted imaging probe. Nucl Med Biol.

[B30] Cheresh DA, Spiro RC (1987). Biosynthetic and functional properties of an Arg-Gly-Asp-directed receptor involved in human melanoma cell attachment to vitronectin, fibrinogen, and von Willebrand factor. J Biol Chem.

